# BMI-1 promotes invasion and metastasis in endometrial adenocarcinoma and is a poor prognostic factor

**DOI:** 10.3892/or.2022.8352

**Published:** 2022-06-20

**Authors:** Jing Yu, Ling Chen, Zhenhua Bao, Ying Liu, Guohong Liu, Fengling Li, Lon Lianqin Li

Oncol Rep 43: 1630–1640, 2020; DOI: 10.3892/or.2020.7539

Following the publication of the above paper, an interested reader drew to the authors’ attention that, in Fig. 5 on p. 1637, the data panel selected to represent the ‘Ishikawa/+BMI-1 siRNA/Invasion’ experiment in Fig. 5A appeared to overlap with the data shown for the ‘JEC/+BMI-1 siRNA/Migration’ panel in Fig. 5C, such that they were potentially derived from the same original source.

The authors have re-examined their original data, and realize that the data panel selected for the ‘Ishikawa/+BMI-1 siRNA/Invasion’ experiment in Fig. 5A was inadvertently selected incorrectly. The corrected version of Fig. 5, showing the correct data for the ‘Ishikawa/+BMI-1 siRNA/Invasion’ experiment in Fig. 5A, is shown below. Note that this error did not affect the overall conclusions reported in the study. The authors are grateful to the Editor of *Oncology Reports* for granting them the opportunity to publish this Corrigendum; furthermore, they apologize for any inconvenience caused to the readership of the Journal.

## Figures and Tables

**Figure 5.**
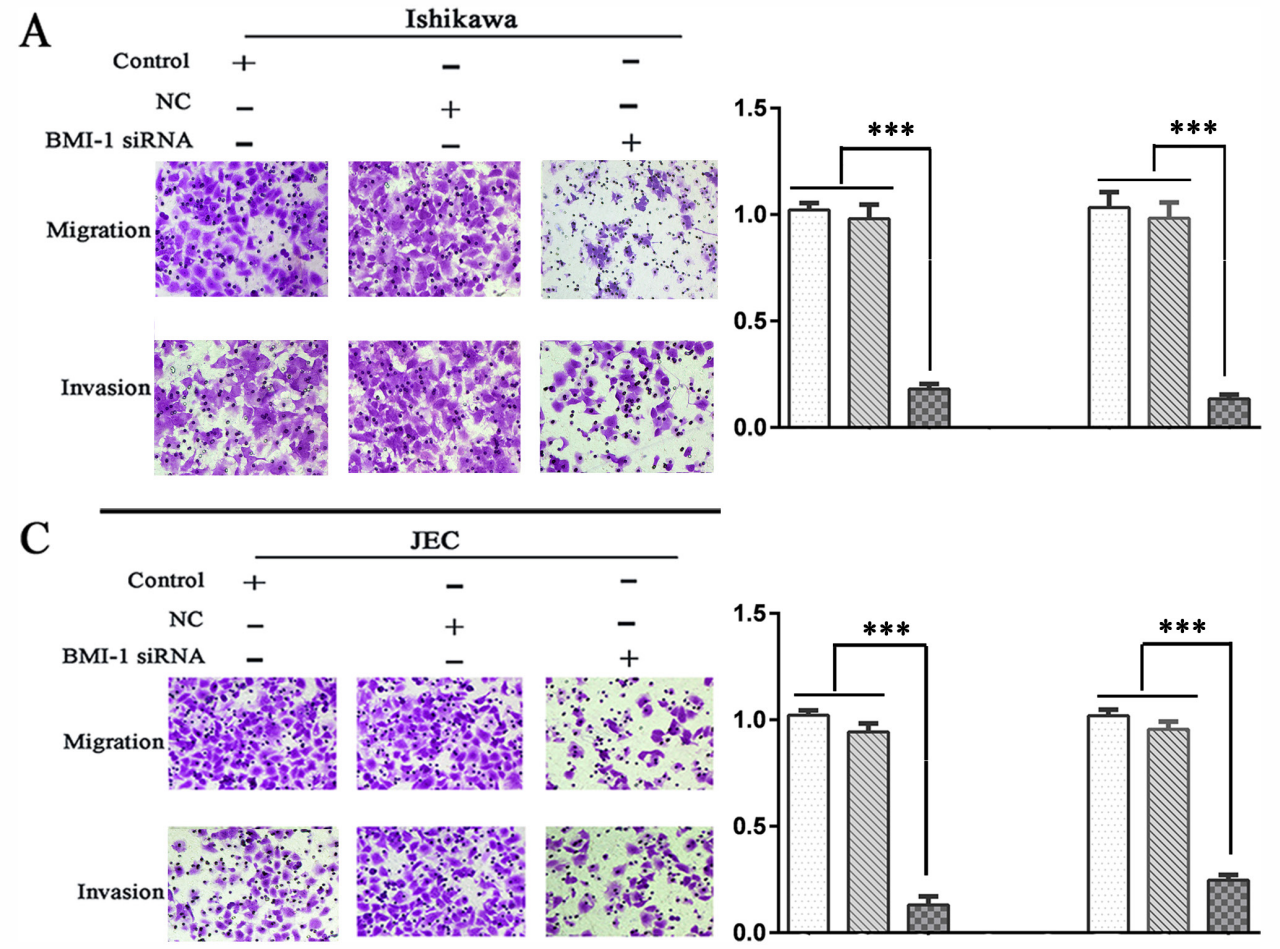
Effect of BMI-1 on migration and invasion of endometrial adenocarcinoma cells. (A) Representative images and (B) quantitative analysis of migration and invasion in Ishikawa cells. (C) Representative images and (D) quantitative analysis of migration and invasion in JEC cells. Magnification, ×200. ***P<0.01. BMI-1, B-lymphoma Mo-MLV insertion region 1; si-RNA, small interfering RNA; NC, negative control.

